# A Randomized Controlled Trial to Evaluate the Role and Efficacy of Oral Glutamine in the Treatment of Chemo-radiotherapy-induced Oral Mucositis and Dysphagia in Patients with Oropharynx and Larynx Carcinoma

**DOI:** 10.7759/cureus.4855

**Published:** 2019-06-07

**Authors:** Shuchita Pathak, Tej P Soni, Lalit M Sharma, Nidhi Patni, Anil K Gupta

**Affiliations:** 1 Radiation Oncology, Bhagwan Mahaveer Cancer Hospital and Research Centre, Jaipur, IND; 2 Medical Oncology, Bhagwan Mahaveer Cancer Hospital and Research Centre, Jaipur, IND; 3 Surgical Oncology, Bhagwan Mahaveer Cancer Hospital and Research Centre, Jaipur, IND

**Keywords:** glutamine, radiotherapy, mucositis, concurrent chemoradiation, dysphagia

## Abstract

Aim: To determine the role and efficacy of oral glutamine in the treatment of chemo-radiotherapy-induced oral mucositis and dysphagia in patients with carcinoma of the oropharynx and larynx. The primary objective of this study was to compare the incidence and severity of oral mucositis and dysphagia between the glutamine group (oral glutamine, along with concurrent chemo-radiotherapy) and the control group (concurrent chemo-radiotherapy alone, no glutamine). Secondary objectives were to compare the time to onset of oral mucositis and dysphagia, the incidence of treatment breaks (more than three consecutive radiation fractions missed), and significant weight loss (more than 3 kilograms of weight loss from the baseline) between the two groups.

Method: A total of 60 patients with locally advanced carcinoma of the oropharynx and larynx (Stage III - IV) who were receiving concurrent chemo-radiotherapy were randomised to the glutamine group (study arm, n = 30) and the non-glutamine group (control arm, n = 30). All patients were treated by radiotherapy to a total dose of 70 Gy in 35 fractions, along with concurrent weekly injections of cisplatin (40 mg/m^2^) chemotherapy. Patients in the study arm received 10 gm of oral glutamine two hours before radiotherapy (five days a week during the seven-week course of radiotherapy). In the control arm, glutamine was not given to patients during the chemo-radiotherapy treatment. All patients were assessed for oral mucositis, dysphagia, weight loss, and compliance/gap in the treatment (greater than three consecutive days of radiation missed) until the completion of chemo-radiotherapy. Grading of oral mucositis and dysphagia was done as per the National Cancer Institute, Common Terminology Criteria for Adverse Events version 4.03.

Results: Out of 60 patients, 56 patients (93.33%) completed the full course of chemo-radiotherapy treatment. Twenty-seven patients (96.43%) in the control arm developed Grade III oral mucositis compared to only 12 patients (42.83%) in the glutamine arm (p < 0.001). Twenty-six patients (93%) in the control arm developed Grade III dysphagia compared to only 11 patients (39%) in the glutamine arm (p < 0.001). Glutamine significantly decreased the incidence and severity of mucositis and dysphagia. Glutamine delayed the onset of mucositis and dysphagia. As severe dysphagia was more prevalent in the control arm, feeding by Ryle’s tube was required in 17 patients (56.67%) in the control arm versus only in eight patients (26.67%) in the glutamine arm (p = 0.03). Significant weight loss during the treatment was seen in all patients of the control arm (100% patients) compared to only 71% of the patients in the glutamine arm (p = 0.004). In the control arm, 46.67% patients had treatment interruption (gap) compared to 16.67% in the glutamine arm (p = 0.025). Sixteen patients (53%) from the control arm required admission (inpatient care) in the hospital to manage the treatment-toxicity (mucositis and dysphagia) compared to seven patients (23%) from the glutamine arm (p = 0.03).

Conclusion: Glutamine significantly decreased the incidence and severity of chemo-radiotherapy-induced oral mucositis and dysphagia. It delayed the onset of oral mucositis and dysphagia, improved the compliance to the chemo-radiotherapy treatment, and reduced the requirement for hospitalization for the management of treatment-induced toxicities in patients with locally advanced carcinoma of the oropharynx and larynx.

## Introduction

Head and neck cancer represents the sixth most common cancer worldwide [1]. It is one of the most common cancers in developing countries like India [2]. In India, it is the most common cancer in males and forms a 30% load of India’s cancer burden [2].

Radiation therapy, along with chemotherapy, forms the main cornerstone and important part of the standard treatment for head and neck cancer. Patients receiving chemo-radiotherapy for head and neck cancer may have significant acute toxicities, including mucositis, skin desquamation, depression and anxiety, cachexia, fatigue, nausea, and vomiting, which can lead to treatment delays, chemotherapy dose deviations, hospitalizations, and poor quality of life [3-4].

Mucositis is a common toxicity of chemo-radiotherapy characterized by the ulceration of oral mucosa causing pain and dysphagia [3-6]. According to various available literature, the incidence of oral mucositis ranges between 80% to 97%, while Grade III and IV mucositis ranges between 40% - 55% depending upon the type of radiotherapy received and the concomitant use of chemotherapy [3-7].

Radiation and/or chemotherapy induce cellular damage, causing the death of the basal epithelial cells and generating free radicals [5-7]. This up-regulates pro-inflammatory cytokines, especially TNF-α, which causes inflammation and ulceration [5-7]. Regimens for the prevention of radiation-induced mucositis include anti-inflammatory drugs, antimicrobial substances, biological response modifiers, and cytoprotective compounds [3-7]. Glutamine has also shown promising results for the alleviation of chemotherapy and radiotherapy-induced oral mucositis [8]. Glutamine is the most abundant amino acid in our body [8-9]. It is a primary fuel and an essential precursor of nucleotide biosynthesis in rapidly proliferating cells, such as enterocytes, fibroblasts, lymphocytes, and macrophages [8-10]. Therefore, glutamine is classified as a conditionally essential amino acid in these cell types [9-11]. Glutamine also serves as a glutathione synthesis substrate and exhibits antioxidant properties. Glutathione, a byproduct of glutamine metabolism protects against oxidant injury [9]. Glutathione is an antagonist to prostaglandin E2 (PGE 2) production, which is a strong inflammatory mediator [9]. Thus, glutamine may help to decrease mucus membrane injury induced by radiation by altering the inflammatory response [8-11]. When exposed to severe stress, the body fails to synthesize sufficient amounts of this amino acid, resulting in decreased plasma glutamine levels [10-11]. Under these conditions, mucosal immunity is negatively affected and glutamine release from muscle tissues is decreased. Patients with advanced cancer undergoing cytotoxic therapy reportedly develop glutamine deficiency [9-10].

A few small studies have evaluated the role and efficacy of glutamine for the management of chemotherapy and radiotherapy-induced toxicity [8-13]. However, the study design of these clinical trials was not adequate to evaluate the role of glutamine in the prevention or treatment of mucositis in head and neck cancer patients, thereby leading to conflicting results [8-13]. The quality of these studies was insufficient since they included a small patient sample, were poorly designed, and used an inadequate glutamine dose; therefore, they were unable to evaluate the effectiveness of glutamine against mucositis in head and neck cancer patients.

The present study assessed whether glutamine decreases the severity of chemo-radiotherapy-induced mucositis in patients with carcinoma of the oropharynx and larynx. In this study, we aim to evaluate the role and efficacy of oral glutamine for the alleviation of chemo-radiation-induced mucositis and dysphagia.

## Materials and methods

The primary objective of the study was to compare the incidence and severity of oral mucositis and dysphagia between the glutamine group (oral glutamine, along with concurrent chemo-radiotherapy) and the control group (concurrent chemo-radiotherapy alone, no glutamine). Secondary objectives were to compare the time to onset of oral mucositis and dysphagia, the incidence of treatment breaks (more than three consecutive radiation fraction missed), and significant weight loss (more than 3 kilograms from the baseline) between the two groups.

This randomized, controlled trial was conducted in accordance with the Helsinki Declaration at the Bhagwan Mahaveer Cancer Hospital and Research Centre in Jaipur, India. The protocol and informed consent form were reviewed and approved by the Institutional Review Board of Bhagwan Mahaveer Cancer Hospital and Research Centre (reference #BMC 2016/2105). All patients provided written informed consent before undergoing any study-related procedures. The present study was also registered with the Clinical Trials Registry, India (CTRI) (registration number CTRI/2017/02/007772). A total of 60 patients with locally advanced carcinoma of the oropharynx and larynx (Stage III-IV) with inclusion criteria of age > 18 years to less than 70 years and fit for chemo-radiotherapy were randomized into two groups (glutamine group and control group) by using the computer-generated random number method. The sample size was calculated at 80% study power and an alpha error of 0.05 assuming a 62.5% incidence of oral mucositis in the treatment group and 98.7% in the control group as per the results obtained in the study of Pattanayak et al. [14]. Twenty-three patients in each group were required as the sample size, which was further enhanced and rounded off to 30 patients in each group (total: 60 patients) as the final sample size for the present study, expecting a 20% drop out/lost to follow-up attrition. All patients were planned for radiotherapy with intensity-modulated radiotherapy (IMRT) (70 Gy of total radiation at 2 Gy/fraction daily and five fractions/week) with concurrent weekly cisplatin (40 mg/m^2^ weekly) chemotherapy. Patients in the glutamine group received 10 gm of oral glutamine with water daily two hours before radiation, five days a week (excluding Saturday and Sunday) during a seven-week course of chemo-radiotherapy. When patients were unable to swallow due to mucositis and dysphagia, glutamine was administered by Ryle’s tube. In the control group, glutamine was not given to patients during chemo-radiotherapy treatment. All patients were evaluated and reviewed weekly during the course of chemo-radiotherapy. Every week, patients were assessed for oral mucositis, dysphagia, weight loss (significant if > 3 kilograms from baseline), and compliance/gap in the treatment (> 3 consecutive days radiation missed) until the completion of chemo-radiotherapy. Grading of oral mucositis and dysphagia was done as per the National Cancer Institute Common Terminology Criteria for Adverse Events, version 4.03. A complete blood count (CBC) and renal function testing (RFT) were done weekly during the treatment. Continuous variables were summarized as the mean and standard deviation, while nominal/categorical variables were summarized as the proportion (%). Paired/unpaired t-tests were used for the analysis of continuous variable, whereas the Chi-square test/Fisher's exact tests were used for nominal/categorical variables. The Mann-Whitney test and Wilcoxon rank sum test were used for ordinal variables or data not following a normal distribution. A P-value < 0.05 was considered significant.

## Results

A total of 60 patients with locally advanced carcinoma of the oropharynx and larynx were enrolled in the study. All patients were randomized and equally distributed in the glutamine group and the control group. The mean age of the patients was 57 years (range: 40 - 71 years). Fifty-four patients (90%) were male and six patients were female (10%). Thirty-seven patients (61.67%) had carcinoma of the oropharynx, while 23 patients (38.33%) had carcinoma of the larynx.

Fifty-six patients (93.33%) completed the full course of chemo-radiotherapy treatment. Two patients (one patient from each group) died due to ischemic heart disease (myocardial infarction) before completion of the chemo-radiotherapy treatment. Two patients (one patient from each group) did not complete the full radiotherapy treatment. These two patients defaulted after 20 - 25 fractions of radiotherapy treatment.

Baseline patient characteristics (sex, age, primary tumor site) were similar between both groups (Table 1).

**Table 1:** Baseline Patient Characteristics Between Both Groups

Characteristics	Control Group		Glutamine Group	
	n	%	N	%
Age 40 - 50 Years	9	30.00	8	26.67
Age 51 - 60 Years	14	46.67	15	50.00
Age > 60 Years	7	23.33	7	23.33
Male	27	90.00	27	90.00
Female	3	10.00	3	10.00
Primary Oropharynx	18	60.00	19	63.33
Primary Larynx	12	40.00	11	36.67

Oral mucositis

There was a significant difference in the time to onset and severity of oral mucositis in the control arm versus the glutamine arm. All grades of oral mucositis appeared early in the control arm compared to the glutamine arm. After the first week of chemo-radiotherapy, a total of seven patients developed Grade I oral mucositis (p = 0.011). All of these seven patients were from the control arm. After the second week of chemo-radiotherapy, a total of six patients developed Grade II oral mucositis. All of these six patients (20%) were from the control arm (p-value < 0.001). At the fourth week of the treatment, six patients (20.69%) in the control arm developed Grade III oral mucositis compared to only one patient (3.33%) in the glutamine arm (p < 0.001). At the fifth week of treatment, 20 patients (71.43%) in the control arm developed Grade III oral mucositis compared to only five patients (17.24%) in the glutamine arm (p < 0.001). At the sixth week, 26 patients (93%) in the control arm compared to 12 patients (42.83%) in the glutamine arm developed Grade III oral mucositis (p < 0.001). At the seventh week of the chemo-radiotherapy treatment, 27 patients (96.43%) in the control arm developed Grade III oral mucositis compared to only 12 patients (42.83%) in the glutamine arm (p < 0.001) (Table 2). 

**Table 2:** Comparison of the Severity of Oral Mucositis in Both Groups at Week 7

Oral Mucositis at Week 7	Control Group		Glutamine Group		Total	
	n	%	n	%	n	%
Grade 2	1	3.57	16	57.14	17	30.36
Grade 3	27	96.43	12	42.86	39	69.64

Dysphagia

There was a significant difference in the time to onset and the severity of dysphagia in the control arm versus the glutamine arm, in favour of the glutamine arm. All grades of dysphagia appeared early in the control arm compared to the glutamine arm. After the second week of chemo-radiotherapy treatment, a total of four patients (13%) from the control arm developed Grade III dysphagia compared to none in the glutamine arm (p <0.001). At the fourth week of the treatment, 11 patients (38%) from the control arm developed Grade III dysphagia compared to none in the glutamine arm (p < 0.001). At the sixth week, 24 patients (86%) in the control group versus 10 patients (36%) in the glutamine arm developed Grade III dysphagia (p < 0 .001). At the seventh week, 26 patients (93%) in the control arm versus 11 patients (39%) in the glutamine arm developed Grade III dysphagia (p < 0.001) (Table 3).

**Table 3:** Comparison of Severity of Dysphagia in Both Groups at Week 7

Dysphagia at Week 7	Control Group		Study Group		Total	
	n	%	n	%	N	%
Grade 2	2	7.14	17	60.71	19	33.93
Grade 3	26	92.86	11	39.29	37	66.07

Weight loss

All 28 patients (100%) in the control arm compared to only 20 patients (71%) in the glutamine arm developed significant weight loss (more than 3 kilograms weight loss from baseline) during the treatment (p = 0.004) (Table 4).

**Table 4:** Comparison of Significant Weight Loss During the Treatment in Both Groups

Weight Loss	Control Group		Glutamine Group	
	N	%	n	%
Yes	28	100.00	20	71.43
No	0	0.00	8	28.57

Treatment interruption

Forty-one (68.33%) patients completed the treatment in seven week’s scheduled time. Nineteen (31.67%) patients had gap or treatment-interruption (more than three consecutive radiation fractions missed) during radiotherapy due to mucositis or dysphagia. Patients from the glutamine arm had fewer treatment breaks or gaps during the treatment compared to the control arm. Fourteen patients (46.67%) in the control arm versus five patients (16.67%) from the glutamine arm had significant gap or treatment breaks during the chemo-radiotherapy course (p = 0.025) (Table 5).

**Table 5:** Comparison of Treatment Interruption in Both Groups

Treatment Break	Control Group		Glutamine Group		Total	
	N	%	n	%	N	%
Yes	14	46.67	5	16.67	19	31.67
No	16	53.33	25	83.33	41	68.33

The requirement of feeding by Ryle’s tube

The incidence of severe dysphagia occurred more in the control arm compared to the glutamine arm. Similarly, feeding by Ryle’s tube was required more often in patients of the control arm. Feeding by Ryle’s tube was required in 17 patients (56.67%) in the control arm versus only in eight patients (26.67%) in the glutamine arm (p = 0.03) (Table 6).

**Table 6:** Comparison of Requirement of Feeding by Ryle’s Tube Between Both Groups

Feeding by Ryle’s tube	Control Group		Glutamine Group		Total	
	N	%	n	%	N	%
Yes	17	56.67	8	26.67	25	41.67
No	13	43.33	22	73.33	35	58.33

Hospitalization/indoor admission for the management of treatment-induced toxicities

The incidence of severe toxicity was higher in the control arm. More patients from the control arm required admission in the hospital for supportive care and symptomatic treatment of oral mucositis and dysphagia compared to the glutamine arm. Sixteen patients (53%) from the control arm required admission to the hospital compared to only seven patients (23%) from the glutamine arm (p = 0.03) (Table 7).

**Table 7:** Comparison of Hospitalization/Indoor Admission of the Patients Between Both Groups

Requirement of Hospitalization/Indoor Admission	Control Group		Glutamine Group		Total	
	n	%	n	%	N	%
Yes	16	53.33	7	23.33	23	38.33
No	14	46.67	23	76.67	37	61.67

## Discussion

The present study demonstrated that glutamine significantly decreased the incidence and severity of chemo-radiotherapy-induced oral mucositis and dysphagia in patients with carcinoma of the oropharynx and larynx. Glutamine also improved compliance with the chemo-radiotherapy treatment.

Oral mucositis is a major concern during chemo-radiotherapy of head and neck cancer. Mucositis causes pain, dysphagia, poor nutrition, poor treatment compliance, and decreased quality of life. Incidence rates of mucositis as high as 80% to 97% have been reported [3-7]. In our study also, Grade III oral mucositis and dysphagia were reported in 62% and 63% patients, respectively. Mucositis, also known as mucosal barrier injury, is a biologically complex process involving several phases [5]. Initially, mucosal injury is induced by oxidative stress and reactive oxygen species (ROS) generated by radiation and chemotherapy. Glutamine protects the mucosal epithelium from the oxidative stress [8]. Glutamine is a precursor of glutathione, a major intracellular antioxidant protecting cells against oxidative stress [9]. Exogenous glutamine administration restores tissue glutathione levels that are depleted after chemotherapy [9-10, 15]. Free and abundant glutamine in the circulation, as well as intracellular pools, is essential for DNA synthesis, cell division, and cell growth, all of which are necessary for wound healing and tissue repair [16]. Glutamine also participates in protein synthesis and extracellular matrix formation [16]. Some studies have shown that glutamine increases collagen synthesis in human fibroblasts by a direct stimulatory effect and as a proline and hydroxyproline residue precursor [17-18]. It also enhances the immune system and is an important fuel for both macrophages and lymphocytes. Intravenous glutamine supplementation reportedly increased IgA production in rats [19]. The aim of this randomized trial was to determine whether oral glutamine decreases the severity of chemo-radiotherapy-induced acute toxicities in carcinoma of the oropharynx and larynx patients.

Leitao et al. showed that glutamine or alanyl-glutamine accelerated mucosal remodeling from 5-fluorouracil-induced oral mucositis by increasing glutathione stores in hamster mucosa [15]. Nose et al. demonstrated that bolus enteral glutamine prevented cisplatin-induced intestinal mucosal injury in rats, possibly resulting in increased intracellular glutathione [20]. Several clinical studies have shown the protective effects of glutamine on the mucosal epithelium [21-23].

Topkan et al. reported that oral glutamine decreased the incidence and duration of acute radiation-induced esophagitis in non-small cell lung cancer patients treated with radiotherapy [21]. In a meta-analysis of 234 patients from five clinical studies, glutamine showed a statistically significant benefit with respect to reducing the risk and severity of radiation-induced oral mucositis compared to either placebo or no treatment (risk ratio 0.17, 95% CI 0.06 - 0.47) [24].

Cerchietti et al., in a double-blinded, placebo-controlled trial, compared 14 patients who received intravenous L-alanyl-L-glutamine during chemo-radiotherapy of head and neck cancer versus 15 patients in the placebo group [22]. The number of patients with severe objective mucositis (objective mucositis score (OMS) > 1.49) was higher in the placebo group compared with the L-alanyl-L-glutamine group (67% vs. 14%, p = 0.007) [22]. Pattanayak et al. [14], in a randomized controlled trial of 162 patients with locally advanced head and neck cancer treated with concomitant chemoradiation, found a significant difference in the incidence, severity, and onset time of mucositis in favor of the glutamine arm compared to the control arm [14]. They observed 53.1% of patients developed mucositis toward the fifth week in the glutamine arm compared with 55.5% of patients in the control arm at the third week. None in the glutamine arm, compared with 92.35% of patients in the control arm, developed severe Grade III mucositis [14]. 

Tsujimoto et al., in a double-blind, randomized, placebo-controlled trial of 40 patients with head and neck cancer treated with concurrent chemo-radiotherapy, found that glutamine reduced the incidence and severity of oral mucositis [25]. In this study, a maximal mucositis grade of Grade IV was observed in none in the glutamine arm patients versus 25% in the placebo arm patients (p = 0.023). Glutamine significantly decreased the maximal mucositis grade (group G, 2.9 ± 0.3; group P, 3.3 ± 0.4; p = 0.005) and pain score at Weeks 4, 5, and 6 [25]. In a randomized trial of 17 patients with head and neck cancer, Huang et al. found that the mean maximum grade of objective oral mucositis was less severe in the glutamine arm (1.6 versus 2.6) (p = 0.0058) and glutamine reduced the duration of Grade III subjective mucositis [8]. In a randomized study of 32 head and neck cancer patients, Chattopadhyay et al. showed that oral glutamine delayed the development of mucositis. The mean time of onset of mucositis was significantly delayed in patients who received glutamine (p < 0.001). The mean duration of Grade III and IV was significantly less (6.6 days versus 9.2 days) in the glutamine arm [26].

In our study also, glutamine considerably decreased the incidence and severity of mucositis and dysphagia. Glutamine also delayed the onset of mucositis and improved treatment compliance. In our study, 96.43% in the control arm developed Grade III oral mucositis compared to only 42.83% of patients in the glutamine arm (p < 0.001). Twenty-six patients (93%) in the control arm versus 11 patients (39%) in the glutamine arm developed Grade III dysphagia (p < 0.001). As severe dysphagia was more in the control arm, feeding by Ryle’s tube was required in 17 patients (56.67%) in the control arm versus only eight patients (26.67%) in the glutamine arm (p = 0.03)

Weight-loss during the treatment is an important and independent unfavourable prognostic factor [27]. In our study, all patients (100%) in the control arm compared to only 71% of patients in the glutamine arm developed significant weight loss during the treatment (p = 0.004).

Noncompliance with radiation therapy may be an indicator of poor outcomes. Patients who miss radiation therapy sessions during cancer treatment have an increased risk of disease recurrence [28]. A study showed that patients who received radiation therapy as planned had a probability of tumour control 10% - 19% higher compared to patients who interrupted the treatment [28]. These differences were statistically significant for the subgroup of patients with advanced tumours (Stage N2-3), patients undergoing concomitant chemo-radiotherapy, and postoperative patients. The major cause for interrupting radiation therapy was the severity of side-effects and intercurrent disease [28]. Preventing measures that minimize treatment breaks are thus fundamental and essential. In our study, patients in the glutamine group had better compliance with the treatment. Fourteen (46.67%) patients in the control arm had treatment interruption to manage the toxicities comparing to only five (16.67%) in the glutamine arm.

In our study, more patients from the control arm required hospitalisation/admission to the hospital for supportive care and symptomatic treatment of oral mucositis and dysphagia compared to the glutamine arm. Sixteen patients (53%) from the control arm required admission in the hospital compared to only seven patients (23%) from the glutamine arm (p = 0.03). Less admission or hospitalisation during chemo-radiotherapy may result in better utilisation of available resources and cost-effective treatment.

In summary, oral glutamine decreased the chemo-radiotherapy-induced severe oral mucositis and dysphagia. We recognize the limitations of our study as it was a single-centre-based study with a small number of patients. There may be bias in the results as blinding/masking during randomization was not designed in this study and investigators, as well as patients, were aware of the glutamine treatment in the study group. Larger, multicentric, and randomized double-blinded studies are recommended to prove the findings of our study. 

## Conclusions

In conclusion, the present study demonstrated that glutamine significantly decreased the incidence and severity of chemo-radiotherapy-induced oral mucositis and dysphagia in patients with carcinoma of the oropharynx and larynx. In our study, glutamine significantly delayed the onset of oral mucositis and dysphagia. Glutamine improved the compliance to chemo-radiotherapy treatment and reduced the chances of hospitalisation for the management of treatment-induced toxicities.
